# Ethnic Specific body fat percent prediction equation as surrogate marker of obesity in Ethiopian adults

**DOI:** 10.1186/s41043-021-00224-3

**Published:** 2021-04-09

**Authors:** Makeda Sinaga, Melese Sinaga Teshome, Tilhun Yemane, Elsah Tegene, David Lindtsrom, Tefera Belachew

**Affiliations:** 1 Department of Nutrition and Dietetics, Faculty of Public Health, Institute of Health, Jimma University, Jimma, Ethiopia; 2 Department of Laboratory and Biomedical Sciences, Faculty of Health Sciences, Institute of Health, Jimma University, Jimma, Ethiopia; 3 Department of Internal Medicine, Faculty of Medicine, Institute of Health, Jimma University, Jimma, Ethiopia; 4 Department of Sociology, Brown University, Providence, USA

**Keywords:** Body fat percent, Prediction, Equation, Ethiopia, Adults

## Abstract

**Background:**

Application of advanced body composition measurement methods is not practical in developing countries context due to cost and unavailability of facilities. This study generated ethnic specific body fat percent prediction equation for Ethiopian adults using appropriate data.

**Methods:**

A cross-sectional study was carried ifrom February to April 2015 among 704 randomly selected adult employees of Jimma University. Ethnic specific Ethiopian body fat percent (BF%) prediction equation was developed using a multivariable linear regression model with measured BF% as dependent variable and age, sex, and body mass index as predictor variables. Agreement between fat percent measured using air displacement plethysmography and body fat percent estimated using Caucasian prediction equations was determined using Bland Altman plot.

**Results:**

Comparison of ADP measured and predicted BF% showed that Caucasian prediction equation underestimated body fat percent among Ethiopian adults by 6.78% (*P* < 0.0001). This finding is consistent across all age groups and ethnicities in both sexes. Bland Altman plot did not show agreement between ADP and Caucasian prediction equation (mean difference = 6.7825) and some of the points are outside 95% confidence interval. The caucasian prediction equation significantly underestimates body fat percent in Ethiopian adults, which is consistent across all ethnic groups in the sample. The study developed Ethnic specific BF% prediction equations for Ethiopian adults.

**Conclusion:**

The Caucasian prediction equation significantly underestimates body fat percent among Ethiopian adults regardless of ethnicity. Ethiopian ethnic-specific prediction equation can be used as a very simple, cheap, and cost-effective alternative for estimating body fat percent among Ethiopian adults for health care provision in the prevention of obesity and related morbidities and for research purposes.

## Introduction

Measurement of body fat percent (BF%) is useful to evaluate the effect of exercise and dietary interventions in weight loss programs [1]. Although body mass index (BMI) is a simple tool used to identify the problem of obesity and risk of metabolic diseases [2], studies indicated that there are ethnic and racial differences in the utility of BMI in predicting BF% [3–6]. One of the drawbacks is that BMI cannot differentiate two individuals with the same weight but different BF% [7–9].

This makes the validity and reliability of international BMI cutoff for the determination of body composition among different ethnic groups questionable. A meta-analysis showed that international BMI cutoff was not able to detect excess BF% in half of the individuals [10]. It has been reported that measurement of BF% gives a more precise estimation of body composition that BMI due to differences in body fat distributions among the different ethnic groups [11]. 

Several studies recommended BF% to be a valid biomarker of obesity and risk of chronic non-communicable diseases [10, 12–16]. Since the past few decades the magnitude of non-communicable diseases (NCDs) is increasing in developing countries since the past few decades [17–19]. World Health Organization (WHO) projected that by 2030, the prevalence of chronic non-communicable diseases oversteps that of communicable diseases in sub-Saharan African countries including Ethiopia [18]. In Ethiopia, the prevalence of cardiovascular diseases (CVD) increased dramatically in the past few years with 31.5% of men and 28.9% of women in Addis Ababa having high blood pressure, indicating a “silent epidemic” of CVDs [20] accounting for over a third of deaths [21]. In Ethiopia, the problem is expected the worst scenario due to high prevalence of early life malnutrition associated with organ stunting [22].

Active surveillance of such epidemiologic transmission is required to prevent the double burden of both NCDs and infectious diseases [23]. Use of advanced methods of measuring biomarkers of obesity including body fat percent is impratical as they are too expensive for routine service use, require qualified personnel, and are not portable for use at the community level [2, 24].

Even though BF% prediction equation was developed from body mass index using advanced techniques [11], the relationship and BMI and body fat percent varies based on ethnic backgrounds [25]. A meta-analysis showed that BF% generated using Caucasian prediction equation significantly underestimated BF% among Ethiopians, Thais, and Polynesians [5], which was hypothesized to be due to the differences in body build. However, the sample of Ethiopians involved in the study was very small making the estimations less reliable. In this study we set out to develop ethnic specific BF prediction equation and determine the agreement between the body fat percent measured by air displacement plethysmography and the Caucasian prediction equation among Ethiopian adults.

## Methods and materials

The study was carried out among 704 employees of Jimma University who were selected randomly using their payroll as a sampling-frame. The university is located 357 km away from Addis Ababa in the southwest direction. It has six colleges and two institutes housing a total of 1341 academic and 5444 administrative staff. The sample size was calculated for developing cutoff values for obesity in the same study participants was used for this analysis. The study participants were selected from Jimma University due to high ethnic diversity compared to the general community in the surrounding. Administrative and academic staffs actively working and were not away for more than one week during recruitment period were considered for inclusion into the study.

Staff members with physical disability including deformity (kyphosis, scoliosis), pregnant women, and limb deformity that prevents them to stand erect and those who were seriously ill during the study period were excluded.

## Measurements

WHO STEPS Questionnaire [26] was used to collect the data after adaptation to the local context. A stepwise approach was followed to collect socio-demographic data, anthropometric measurements, and body fat percent. Five clinical nurses used as data collectors and supervisors were given a 5-days intensive training on the interviewing approach, anthropometric measurements, and data recording before the actual data collection. All the measurements and interviews were done under close supervision.

### Anthropometry

Body-weight was measured to the nearest 0.1 kg with a digital scale of the air displacement plethysmography (COSMED, Rome, Italy). The validity of the scale was checked using an object of a known weight every morning. Height was measured with an adjustable portable stadiometer which was accurate to 0.1 cm in a private place with the study participants wearing light clothing and their heads positioned at the Frankfert Plane and the four points (heel, calf, buttocks, and shoulders) touching the vertical stand and their shoes taken off. Before starting the measurements, the stadiometer was checked using a rod of known length. Body mass index (BMI) was calculated by dividing weight in kilograms by height in meters squared.

### Body fat percent (BF %)

Body fat percent was determined using air displacement plethysmography (COSMED, Rome, Italy) [27–29] after calibration of the machine for adults. The subjects wore a similar swimming pants and swimming cap covering the hair to prevent air trapping under clothing while all ADP tests were conducted.

At the beginning of each testing day, quality-control procedures were performed. The participants were asked not eat or exercise and drink coffee 4–5 h prior to the test. They were also told not to smoke or drink alcohol within 2 h, not to participate in vigorous/high-intensity weight training 12 h prior the test and were given advice to come after resting. For each participant, age, sex, height, and identifiers (ID) were entered into a computer. A two-step calibration procedure was then performed, first with the empty test chamber and then with a calibration cylinder. While the second calibration step was being performed, the subject was weighed on a calibrated electronic scale. Next, the participant was asked to sit inside the BOD POD chamber and instructed to remain still and continue normal breathing while the body volume was being measured. The measurement took two minutes and ADP was used in this study as the standard reference (± 3%) of BF% [30].

### Data analysis

The questionnaire was checked for completeness by the investigator every day. Data were edited and doubly entered into EpiData version 3.1 and then exported to cleaned and analyzed using SPSS for Windows version 20. Descriptive analysis was conducted to describe the study subjects. Multivariable linear regression was used to determine the relationship between BF% measured with ADP as the dependent variable and BMI, age, and sex as independent variables after checking all assumptions.

First, body fat percent prediction equations were generated for the different Ethnic groups. Differences between measured BF% and the one predicted Caucasian equation were tested for significance by the paired two-sided Student’s *t* test. All results were expressed as means with their standard deviations.

A Bland Altman plot was generated to determine the agreement between the BF% measured using ADP and BF% predicted by the E Caucasian prediction equations. The difference between measured BF% and BF% predicted by the Caucasian prediction equation was plotted on the *Y*-axis against its average on the *X*-axis. According to the recommendation, 95% of the data points should lie within the ± 1.96SD of the mean difference and mean difference should be at 0 point on the graph [31].

### Ethical consideration

Before data collection, the study was approved by the Institutional Review Board of Jimma University College of Health Sciences. Written informed consent was also obtained from the study participants after the purpose of the study was clearly explained to all study participants. The right of study participants to refuse participation or withdraw from the study at any point was respected. All data were kept confidential and the study participants were informed that the information they gave will not be disclosed to the third person. To assure complete confidentiality, other identifying information including name were not recorded on questionnaire.

## Results

Out of 705 samples planned, a total of 704 employees of Jimma University were involved in the study giving a response rate of 99.85%. A little over half (56.4%) were females and a larger proportion (38.2%) of the study participants were in the age group between 20 and 30 followed by those in the age group of 31 and 40 years. The mean age (± SD) was 34.7(± 9.5) years for males and 36.5 (± 9.2) years for females. A large proportion of the study participants was Oromo (36.2%) by ethnicity followed by Amhara (30.3%).

The mean height (± SD) was 171.8 (± 13.4) cm for males and 157.1 (± 8.5) cm for females. The mean weight was 66.0 (± 11.7) kg for males and 62.3 (± 12.9) kg for females. The mean BMI was higher for females compared to males (25.3 kg/m^2^ vs 22.5 kg/m^2^). The measured body fat mass percent (mean ± SD) was 38.5% for females and 23.9% for males (Table 1).

**Table 1:** Background and anthropometric characteristics of the study participants (*n* = 704)

Characteristics	*n*	Percent
Sex		
Female	397	56.4
Male	307	43.6
Ethnic groups		
Oromo	255	36.2
Amhara	213	30.3
Gurage	38	5.4
Kefa	50	7.1
Others (Sidama, Wolaita, Tigre)	48	6.8
Dawero	57	8.1
Yem	43	6.1
Age group (years)		
20–30	269	38.2
31–40	250	35.5
≥ 41	185	26.3
		Mean (SD)
Height (cm)		
Male	307	171.8 (13.4)
Female	397	157.1 (8.5)
Weight (kg)		
Male	307	66.0 (11.7)
Female	397	62.3 (12.9)
BMI (kg/m^2^)		
Female	397	25.3 (5.1)
Male	307	22.5 (3.9)
Total	704	24.1 (4.8)
ADP measured body fat mass fat % (mean ± SD)		
Female	397	38.5 (10.1)
Male	307	23.9 (9.2)

*SD* standard deviation, *kg* kilogram, *ADP* air displacement plethysmography

Comparison of ADP measured and predicted BF% showed that Caucasian prediction equation underestimated body fat percent among Ethiopian adults by 6.78% (*P* < 0.0001). This finding is consistent across all age groups and ethnicities in both sexes. The underestimation was higher among males (7.21%, *P* < 0.0001) than females (6.45%, *P* < 0.0001). There was maximum underestimation among Gurage ethnicity (8.53, *P* < 0.0001) followed by Amhara (7.92%, *P* < 0.0001), while the lowest underestimation was recorded among Yem (4.31) followed by Dawero ethnic groups (5.08%, *P* < 0.0001). The difference between measured and estimated BF% using Caucasian prediction equation showed a significant variation with age. The highest difference(7.59%, *P* < 0.0001) was observed in the age group of 31–40 years followed by those in the age group above 40 years (7.18%, *P* < 0.0001) (Table 2).

**Table 2:** Differences between measured and predicted body fat percentages using Caucasian prediction equations among Ethiopian Adult employs of Jimma University

Variables	*n*	Measured body fat % − predicted body fat % using Caucasian prediction equation^a^			
		Measured, body fat%	Caucasian equation, predicted body fat %	Difference	P*
Sex					
Male	307	23.86	18.7764	7.2153	< 0.0001
Female	397	38.47	33.3513	6.4478	< 0.0001
Total	704	32.10	26.99550	6.7825	< 0.0001
Ethnicity					
Oromo	255	29.02	23.9985	6.8420	< 0.0001
Amhara	213	36.56	30.1531	7.9221	< 0.0001
Gurage	38	35.05	28.1747	8.5323	< 0.0001
Kefa	50	33.96	29.7953	5.7637	< 0.0001
Dawero	57	31.49	28.1145	5.0774	< 0.0001
Yem	43	29.12	26.5283	4.3102	< 0.0001
Others^b^	48	27.77	24.1443	5.3252	< 0.0001
Total	704	32.10	26.9955	6.7825	< 0.0001
Age group					
20–30	269	25.55	21.43	5.7647	< 0.0001
31–40	250	35.71	29.62	7.5860	< 0.0001
≥ 41	185	36.75	31.54	7.1767	< 0.0001
Total	704	32.10	27.00	6.7825	< 0.0001

^a^Caucasian prediction equation: body fat percent (BF %) = (1.294 × BMI) + (0.0.20 × Age) − (11.4 × Gender) − 8.0, where sex (male = 1, female = 0) [5]

*Paired *t* test

^b^Others = Sidama, Wolaita, and Tigre

**Fig. 1:**
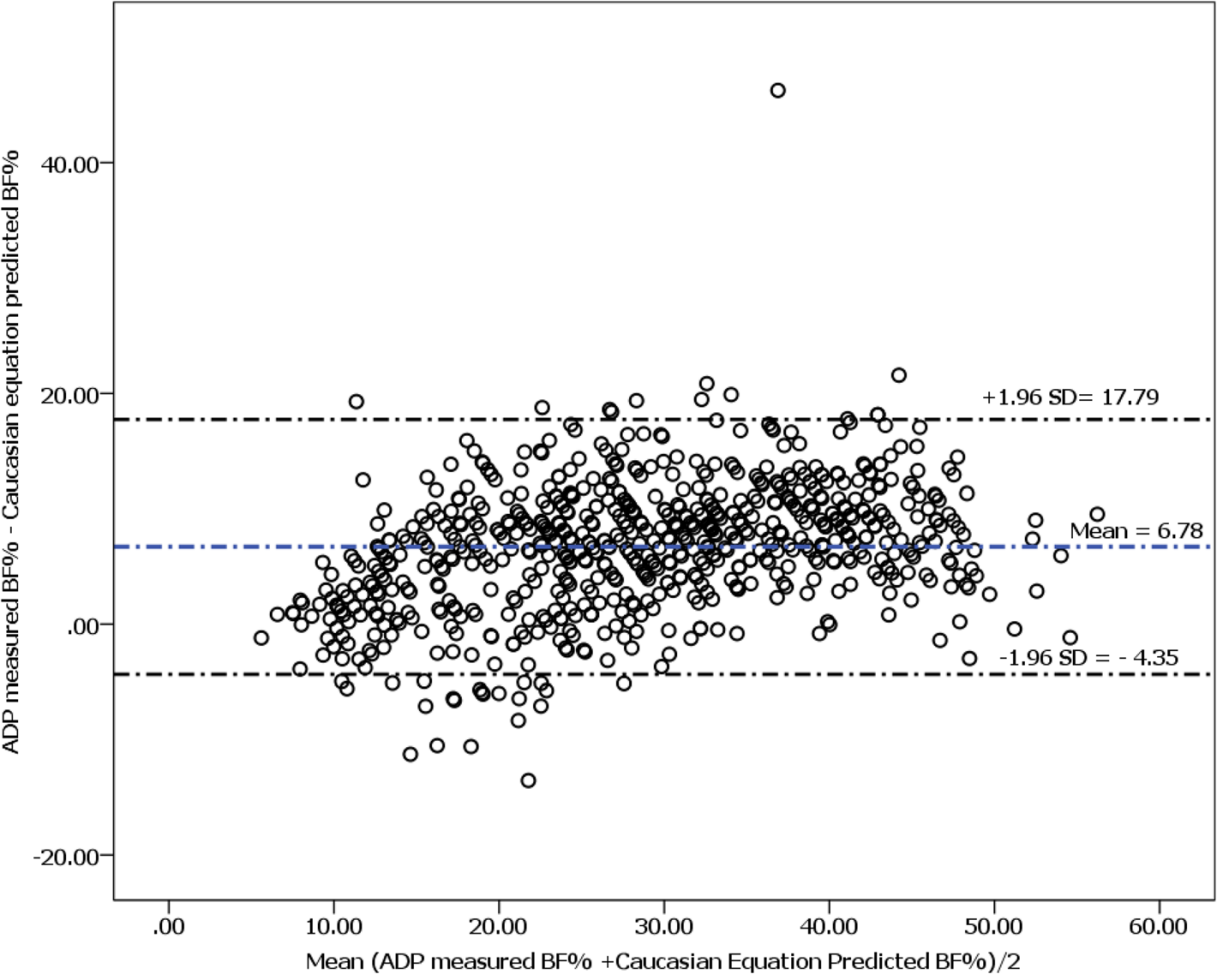
Bland Altman plot showing the agreement between ADP measured body fat percent and body fat percent estimated using Caucasian prediction equation among Ethiopian adults

As shown in Fig. 1, there is poor agreement between the BF% using the Caucasian prediction equation as the mean difference is different from zero (mean difference = 6.7825), and some of the points are outside the 95% confidence interval.

The ethnic specific body fat percent prediction equation were developed using linear regression based on body mass index, age, and sex as as presented in Table 3.

**Table 3:** Multivariable linear regression model predicting body fat mass percent (BF %) as a dependent variable and body mass index (BMI), age, and sex as independent variables for different ethnic groups of Ethiopia

Sample	*n*	BMI		Age		Sex		Intercept		Adjusted *R*^2^
		*β*	SE	*β*	SE	*β*	SE	*β*	SE	
Oromo	255	1.421	0.085	0.261	0.040	− 11.106	0.794	− 6.340	2.271	0.785
Amhara	213	1.367	0.069	0.213	0.040	− 10.600	0.713	− 2.721	2.104	0.825
Gurage	38	1.575	0.250	0.266	0.101	− 7.974	1.866	− 10.291	5.918	0.751
Kefa	50	1.779	0.234	0.292	0.104	− 10.738	2.111	− 17.622	1.866	0.708
Dawero	57	1.641	0.266	0.156	0.117	− 6.791	2.423	− 10.774	6.879	0.526
Yem	43	1.683	0.172	0.148	0.068	− 9.977	1.646	− 10.961	4.420	0.788
Others^a^	48	1.606	0.172	0.124	0.099	− 8.531	1.646	− 9.265	4.785	0.728

Where sex (male = 1, female = 0)

*BMI* body mass index, *SE* standard error

^a^Others = Sidama, Wolaita

## Discussion

The results demonstrated that Caucasian prediction equation significantly underestimates BF% among Ethiopian adults, which is consistent with the finding reported from a study conducted among adult population of different ethnic groups [5]. It was also reported that body-mass index (BMI) may misclassify subgroups with excess body fat among the different races and genders significantly [3]. Different levels in energy intake and energy expenditure and in body build and body frame could be possible explanations for underestimation of BF % by the Caucasian equation [6]. Differences in body build have also been reported within Caucasian populations [5, 32, 33].

Ethiopian ethnic-specific prediction equation showed the best estimate of BF%. Ethiopian general prediction equation worked well but does not predict BF% for some ethnicities like Amharae and Yem for which there were significant differences between BF% measured by ADP and that estimated using the prediction equation. There was no statistically significant difference (*P* > 0.05) between measured body fat and predicted body fat using ethnic-specific prediction equations. Our findings showed that the Caucasian prediction equation underestimated body fat percent of Ethiopian adults when validated against ADP, which is considered to be an accurate method in healthy adults [34]. The Bland Altman plot [31] also showed that the differences plotted on the *Y*-axis against the average of measured fat and predicted fat on the *X*-axis showed that 95% confidence interval did not include all the points and the mean difference is greater than zero showing the Caucasian prediction equation underestimates body fat percent. Thus, the Ethiopian prediction equation could be used to identify BF% as a modifiable risk factor, manage patients, and develop guidelines for control and prevention of obesity and related non-communicable diseases in Ethiopia.

Subjects with a small body frame are likely to have a relatively lower fat-free mass (due to lower muscle mass) compared to other people of the same body height and hence BMI is likely to underestimate their body fat when the prediction equation developed in subjects with a bigger body build is applied [5, 6, 33]. The reason for the different relationships between body fat and BMI in the different populations is unknown. Apart from differences between dietary patterns and differences in physical activity, variations in body build may be an important contributor [33, 35]. It has been shown that the relation between body fat percent and MBI is curvilinear [36] indicating that BMI underestimates BF% even more for very obese subjects.

Differences in the relative leg length lead to differences in body fat percent for the same BMI such that people with lower sitting height to height ratio (people with longer legs relative trunk) have lower body fat percent [37]. It is known that there are differences in relative sitting height between Caucasians and Blacks, and between Caucasians and Asians, with blacks having relatively longer legs and Asians having relatively shorter legs [35, 38]. Apart from relative leg length, a stocky or slender body build may be one of the explanations for the difference [6]. A stocky person is expected to have more muscle mass compared to a slender person of the same body height [6, 39]. As Ethiopians have a slender body build, the Caucasian equation could underestimate their body fat percent. Thus, for the same BMI, the slender person will have more body fat [38]. Additional anthropometric measures may be necessary to improve the quality of the BMI as an indicator of body fatness among ethnic groups [5, 33, 38].

Due to the unavailability of the standard portable machine for the measurement of BF%, equations such as the one developed by this study are very important to calculate the body fat percent for each ethnicity. Assessment of body fat percent at the community level is important for public health policies to detect the magnitudes of overweight and obesity. In recent years, there have been numerous studies conducted to find out the best anthropometric indicator for detecting body fatness among different population groups [40–44].

The fact that there are ethnic and racial differences in the predictive power of BMI-based equation has been reported [5, 33, 38]. The need for developing ethnic-specific prediction equations has been suggested to avoid such a problem [38]. The findings, have practical implications in the wake of an increasing level of obesity due to the tendency of urbanization, dietary transition, increasing consumption of more processed foods, and transition into a motorized way of life. This needs a simple tool for early detection and public health interventions, as the facilities for determination of body fat precept like ADP are expensive and non-portable, which make them unavailable for day to day service provision. Therefore, the development of body fat percent prediction equation based on locally relevant data is a critical input to such prevention efforts. This is especially important as Ethiopia is aspiring to be a middle-income country by 2025, life expectancy has risen to 64 years, and there is an increasing prevalence of chronic non-communicable diseases related to obesity [45, 46]. Prevention of NCDs will enable the country to reap its demographic dividend by avoiding untimely death and disability among working adults [47].

The current study has the strength of using a large sample size and developing the prediction equation for the different ethnic groups. Although getting a sample of all ethnicities in Ethiopia was difficult given the non-portable nature of the ADP, the study tried to include the ethnicities that constitute the majority of the population.

The fact that the study used local data for the development of a prediction equation and ADP as the gold standard for measuring body fat percent are some of the strengths. Although Ethiopian prediction equation was developed using a multiethnic sample (training group) future research should validate its performance in estimating body fat percent in a different sample of multiethnic composition (testing group).

## Conclusion

The Caucasian prediction equation significantly underestimates body fat percent among Ethiopian adults. As advanced methods such as ADP are neither available nor portable for use at the community level, the Ethiopian ethnic specific prediction equation can be used as a very simple cheap effective alternative for estimating body fat percent among Ethiopian adults for health care provision and research purposes.

## Data Availability

The datasets used and/or analyzed during the current study are available from the corresponding author on reasonable request.
